# Metabolomics Analysis of Different Varieties of *Pouteria caimito* Fruit Based on UHPLC-MS/MS

**DOI:** 10.3390/foods15162855

**Published:** 2026-08-15

**Authors:** Haijie Huang, Li Zhao, Zhikai Wang, Yang Qiao, Huidong Deng, Yingjun Ye, Kaili Ding, Xuejie Feng, Yijun Liu

**Affiliations:** 1 Hainan Provincial Key Laboratory for Tropical Fruit Biology, Institute of Tropical Fruit Trees, Hainan Academy of Agricultural Sciences, Haikou 571100, China; huanghaijie1979@163.com (H.H.); wangzhikai.stu@yangtzeu.edu.cn (Z.W.); qiaoyang0701@163.com (Y.Q.); denghuidong@hnaas.org.cn (H.D.); 17327767270@163.com (Y.Y.); 2 Hainan Key Laboratory of Storage & Processing of Fruits and Vegetables, Agricultural Products Processing Research Institute, Chinese Academy of Tropical Agricultural Sciences, Zhanjiang 524001, China; dkl199888@163.com; 3 Tropical Biodiversity and Bioresource Utilization Laboratory, College of Science, Qiongtai Normal University, Haikou 571100, China; zhaoli@mail.qtnu.edu.cn

**Keywords:** *Pouteria caimito*, metabolomics, UHPLC-MS/MS, differential metabolites, KEGG pathway, variety specificity

## Abstract

To investigate the differences in pulp metabolites among four varieties of *Pouteria caimito* fruit (JZL1, JZL3, JZL5, and JZL6), ultra-high performance liquid chromatography-tandem mass spectrometry (UHPLC-MS/MS) was employed for the separation and identification of metabolites. A total of 1285 metabolites were identified, including 648 in positive ion mode and 637 in negative ion mode. At the class level, carboxylic acids and derivatives (14.32%) and organooxygen compounds (11.83%) accounted for the highest proportions; at the subclass level, amino acids, peptides, and analogues (12.53%) as well as carbohydrates and carbohydrate conjugates (9.49%) were the main categories. PLS-DA analysis revealed significant differences in metabolite profiles among the four varieties, with numerous up-regulated and down-regulated metabolites in each comparison group, and some metabolites showed fold changes > 10 or <0.1. KEGG pathway enrichment analysis further indicated that differential metabolites were primarily enriched in pathways such as ABC transporters, citrate cycle (TCA cycle), starch and sucrose metabolism, and linoleic acid metabolism. This study provides an important metabolomic basis for variety identification, nutritional quality evaluation, and functional component development of *Pouteria caimito* fruit.

## 1. Introduction

*Pouteria caimito* Radlk., commonly known as yellow crystal fruit, golden fruit, or honey egg fruit, is a perennial evergreen tree of the Sapotaceae family. It is native to the upper Amazon region of South America and has been widely introduced and cultivated in Latin America, Australia, Southeast Asia, as well as tropical and south subtropical regions of China. Ripe *Pouteria caimito* fruit is golden and translucent, with milky white, semi-transparent pulp that is sweet, juicy, and characterized by a complex vanilla and milky aroma, making it highly popular in the fresh fruit market [1,2]. *Pouteria caimito* fruit is not only rich in nutrients such as proteins, carbohydrates, dietary fiber, calcium, phosphorus, iron, and various vitamins [3,4,5], but also contains abundant phenolic compounds (e.g., catechin and its derivatives), flavonoids, organic acids, volatile organic compounds (VOCs), and fatty acids including palmitic acid, elaidic acid, and linoleic acid [6,7]. Long-term consumption is believed to promote digestion, relieve constipation, enhance immunity, and nourish the skin, earning it the reputation of “bird’s nest among fruits”. However, although the fruit has been introduced to China for nearly three decades, the industry is still in its early stages. Most large-scale plantings are seedling-grown trees, leading to significant variations in ripening periods and fruit quality among individual trees, which severely restricts standardized production and industrial upgrading. Therefore, clarifying the quality differences among different *Pouteria caimito* fruit varieties and their underlying metabolic basis is of great theoretical and practical significance for promoting variety improvement, guiding standardized industrial production, and facilitating the precise screening of high-quality germplasm resources.

Varietal differences and cultivation methods significantly affect the quality of *Pouteria caimito* fruit [8,9]. With increasing market demand for *Pouteria caimito* fruit, its germplasm resources are continuously expanding. Eighteen germplasm resources (J1–J18) were evaluated by Jiang et al. [10], and it was found that J7 exhibited excellent comprehensive performance (single fruit weight of 308 g, edible rate of 65.07%, soluble sugar of 13.51%, and soluble solids of 15.9%). J18 was observed to have the largest single fruit weight (338.8 g) but average quality, and thus it was considered suitable as a large-fruit breeding material. J1 was found to possess high nutritional quality but relatively small fruit size, making it suitable as a small-fruit breeding material. The introduced new varieties, ‘Dajinsha’ (thick-skinned, storage-tolerant, cold-resistant) and ‘Mitang’ (non-oxidizing flesh, 18 °Brix), were compared by Fang et al. [2], and it was found that three-year-old trees produced more than 50 kg per year, with traits superior to those of local common varieties. It was discovered by Zhang et al. [11] that greenhouse cultivation helped increase the single fruit weight, longitudinal diameter, and transverse diameter of *Pouteria caimito* fruit, and also improved the soluble solids and total sugar content of ripe fruits. A significant correlation was observed between fruit skin color difference and fruit traits. The botanical characteristics of 11 *Pouteria caimito* fruit germplasm resources, including HJG056, were evaluated by Zhou et al. [8], and it was found that great variations in botanical traits and fruit quality were exhibited among the germplasm resources, indicating that rich genetic diversity was possessed. For example, germplasm HJG151 was observed to have vigorous overall tree growth, with ripe fruits being round and golden, an average single fruit weight as high as 276.47 g, and an edible rate of 67.64%; HJG134 was found to have the highest contents of vitamin C (0.50 mg/g) and protopectin (0.98%). However, insufficient attention has been paid to the fine differences in internal chemical composition among different *Pouteria caimito* fruit varieties in existing studies, necessitating the urgent application of high-throughput, high-resolution modern analytical techniques for molecular-level elucidation.

Metabolomics enables the qualitative and quantitative analysis of small-molecule metabolites in biological samples and has been widely applied in plant physiology, food science, and agricultural breeding [12,13]. In particular, it plays a critical role in fruit quality research. In terms of flavor quality, it was found that among 477 metabolites in wild bayberry during ripening, 130 changed significantly. In ripe fruits, metabolites such as flavonoids and terpenoids were up-regulated, while amino acids, phenolic acids, and organic acids associated with sourness were down-regulated, directly affecting flavor transformation [14]. A study on yellow passion fruit revealed 154 common differential metabolites across five ripening stages. The accumulation of sugars and fatty acids increased significantly at specific stages, whereas citric acid decreased notably. L-Sorbose and 5-hydroxyindole-3-acetic acid were identified as potential flavor biomarkers positively correlated with the sugar/acid ratio and negatively correlated with titratable acidity [15]. Another study elucidated the close association of amino acids, sugars, and anthocyanins with flavor formation in wild strawberries, highlighting the nutritional and antioxidant value of these compounds [16]. Various polyphenols (e.g., flavonol glycosides, phenolic acids) have been identified in pears and found to be crucial for fruit quality and health benefits [17]. A comparative metabolomic analysis of four pineapple varieties showed that 231 common metabolites (e.g., sucrose, glucose, citric acid) exhibited no significant differences; however, differentially accumulated metabolites (DAMs) were mainly concentrated in phenolic acids, flavonoids, amino acid derivatives, and alkaloids, with each variety displaying distinct accumulation patterns. For example, ‘Tainong No. 23’ was rich in branched-chain amino acids and various flavonoids and phenolic acids [18]. Collectively, these studies demonstrate that metabolomics is an effective strategy for deciphering fruit metabolic differences, screening quality markers, and elucidating the mechanisms underlying flavor and nutritional formation.

However, research on *Pouteria caimito* fruit remains quite limited both domestically and internationally to date, lagging far behind that on other major tropical fruits. A small number of reports have focused on the introduction of new varieties, the determination of conventional nutrient contents in fruits, and simple comparisons of seedling-derived lines [2,19], while no study has yet been reported on the differences in metabolites among different *Pouteria caimito* fruit varieties. In this study, four industrially representative *Pouteria caimito* fruit varieties, JZL1, JZL3, JZL5, and JZL6, were selected as research subjects. Ultra-high performance liquid chromatography-tandem mass spectrometry (UPLC-MS/MS), combined with partial least squares discriminant analysis (PLS-DA), was employed to analyze and compare the metabolites of the four varieties. The aim is to elucidate the quality differences among different *Pouteria caimito* fruit varieties and the key metabolic pathways underlying these differences, thereby providing a solid theoretical and data foundation for the evaluation of *Pouteria caimito* fruit germplasm resources and the discovery of functional components.

## 2. Materials and Methods

### 2.1. Materials

*Pouteria caimito* fruits were purchased from Hainan Golden Palm Ecological Technology Development Co., Ltd. (Haikou, China), including four varieties designated as JZL1, JZL3, JZL5, and JZL6. The experiment was conducted with three biological replicates, each replicate consisting of 10 fruits at the same maturity stage (determined by skin color and firmness). All fruits were collected from five randomly selected trees in the same orchard during a single harvesting period. After harvesting, the fruits were peeled, immediately homogenized, flash-frozen in liquid nitrogen, and subsequently transported to the laboratory with dry ice insulation for analysis.

Reagents: Methanol and acetonitrile (chromatographic grade) were purchased from Sigma (Burlington, MA, USA). Ammonia solution was purchased from Shanghai Aladdin Biochemical Technology Co., Ltd. (Shanghai, China).

### 2.2. Sample Pretreatment

After harvesting, the *Pouteria caimito* fruits were cleaned with purified water, air-dried, and then immediately frozen in liquid nitrogen. The frozen samples were stored at –20 °C until use.

### 2.3. Metabolite Extraction

Metabolite extraction was performed according to the method described by Huang et al. [20]. Samples stored at −20 °C were taken out and slowly thawed at 4 °C. A 1.0 g of each sample was accurately weighed, and a pre-chilled methanol/acetonitrile/water mixture (2:2:1, *v*/*v*/*v*) was added. After vortex mixing, the mixture was ultrasonicated at low temperature for 30 min, followed by standing at −20 °C for 10 min. The mixture was then centrifuged at 14,000× *g* for 20 min at 4 °C, and the supernatant was collected and dried under vacuum. Prior to mass spectrometry analysis, the dried residue was reconstituted in 100 μL of acetonitrile/water solution (1:1, *v*/*v*), vortexed, and centrifuged again at 14,000× *g* for 15 min at 4 °C. Finally, the supernatant was collected for injection analysis.

### 2.4. Metabolite Determination

Metabolite determination was carried out according to the method described by Huang et al. [20].

Chromatographic conditions: A 2 μL aliquot of the sample was placed in an autosampler maintained at 4 °C. Separation was performed on a C18 column at a column temperature of 25 °C, with a mobile phase flow rate of 0.4 mL/min. The gradient elution program for mobile phases A (consisted of 25 mM ammonium and 0.5% formic acid) and B (consisted of methanol) was set as follows: from 0 to 0.5 min, then B changed to 100% linearly from 0.5 to 10 min, 100% B was maintained; from 10 to 12 min. Next, 100% B was linearly decreased 5% from 12.1 to 16 min, and maintained at 5%.

Mass spectrometry conditions: An Agilent 1290 Infinity LC ultra-high performance liquid chromatography system coupled with an AB Triple TOF 6600 mass spectrometer (Foster City, CA, USA) was used. After UHPLC separation, the samples were detected in both positive and negative electrospray ionization (ESI) modes. The ESI source parameters were set as follows: nebulizer gas (Gas1) 60 psi, auxiliary heating gas (Gas2) 60 psi, curtain gas (CUR) 30 psi, ion source temperature 600 °C, ion spray voltage (ISVF) ± 5500 V (set separately for positive and negative modes). The MS scan range was set to mass-to-charge ratio (*m*/*z*) 60–1000 Da. The MS/MS scan range was set to *m*/*z* 25–1000 Da. The accumulation time for MS scan was 0.20 s/spectra, and for MS/MS scan was 0.05 s/spectra. MS/MS data were acquired using information-dependent acquisition (IDA) mode, with selection based on peak intensity threshold. The declustering potential (DP) was set to ±60 V (positive/negative modes), and the collision energy was set to 35 ± 15 eV. The IDA parameters were set as follows: dynamic exclusion of isotopic ions within 4 Da, and 10 fragment spectra were collected per scan.

### 2.5. Data Process

The raw MS data (wiff.scan files) were converted to MzXML files using ProteoWizard MSConvert before importing into freely available MS-DIAL 4.0 software. For peak picking, the following parameters were used: centWave *m*/*z* = 10 ppm, peakwidth = c (10, 60), prefilter = c (10, 100). For peak grouping, bw = 5, mzwid = 0.025, minfrac = 0.5 were used. CAMERA (Collection of Algorithms of MEtabolite pRofile Annotation) was sued for annotation of isotopes and adducts. In the extracted ion features, only the variables having more than 50% of the nonzero measurement values in at least one group were kept. Compound identification of metabolites was performed by comparing of accuracy *m*/*z* value (<10 ppm), and MS/MS spectra with an in-house database established with available authentic standards.

### 2.6. Statistical Analysis

Multivariate statistical analyses, including partial least squares discriminant analysis (PLS-DA), were performed using SIMCA 14.1 (Umetrics, Umeå, Sweden). Permutation tests (200 times) were conducted to validate the model. Student’s *t* test was applied to determine the significance of differences between two groups of independent samples. *p* value < 0.05 were used to screen significant changed metabolites. Pearson’s correlation analysis was performed to determine the correlation between two variables. Commercial databases including was used for pathway enrichment analysis.

## 3. Results and Discussion

### 3.1. Separation and Identification of Metabolites in Pouteria caimito Fruit

**Figure 1:**
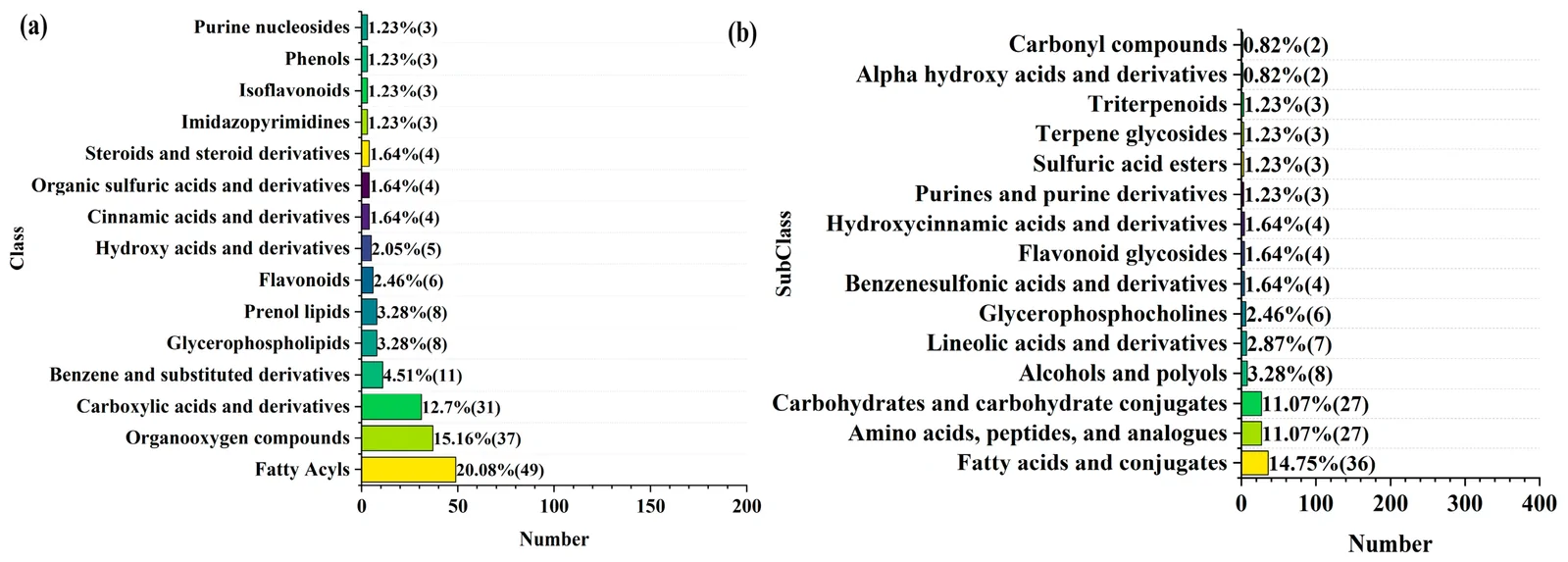
Metabolite classification in *Pouteria caimito* fruit pulp at the Class (**a**) and SubClass (**b**) levels.

As shown in Tables S1 and S2, a total of 244 metabolites, including usnic acid, fluprostenol, and quinic acid, were identified from the pulp of four *Pouteria caimito* fruit varieties (JZL1, JZL3, JZL5, and JZL6), of which 64 were identified in positive ion mode and 180 in negative ion mode. As illustrated in Figure 1a and Table S3, at the Class level, a total of 37 classes of metabolites were identified in the pulp of *Pouteria caimito* fruit, among which fatty acyls (20.08%) and organooxygen compounds (15.16%) accounted for relatively high proportions, indicating that the pulp is rich in organic acids as well as sugars, alcohols, aldehydes, and other compounds related to flavor and energy metabolism [21,22]. Furthermore, as shown in Figure 1b and Table S4, at the SubClass level, a total of 66 subclasses of metabolites were identified, with fatty acids and conjugates (14.75%), Amino acids, peptides, and analogues (11.07%) accounting for relatively high proportions, highlighting the advantages of *Pouteria caimito* fruit pulp in terms of nutritional value, umami characteristics, and sweetness [23]. The differences between the two classification levels reflect the complementarity of chemical structure-based classification and biological function-based classification. Collectively, these results indicate that the pulp of *Pouteria caimito* fruit is not only rich in basic metabolites such as organic acids and sugars but also contains abundant amino acids and small peptide active components, providing an important basis for in-depth analysis of its flavor quality, nutritional functions, and postharvest metabolic regulation mechanisms.

### 3.2. PLS Analysis of Metabolites in Pouteria caimito Fruit

**Figure 2:**
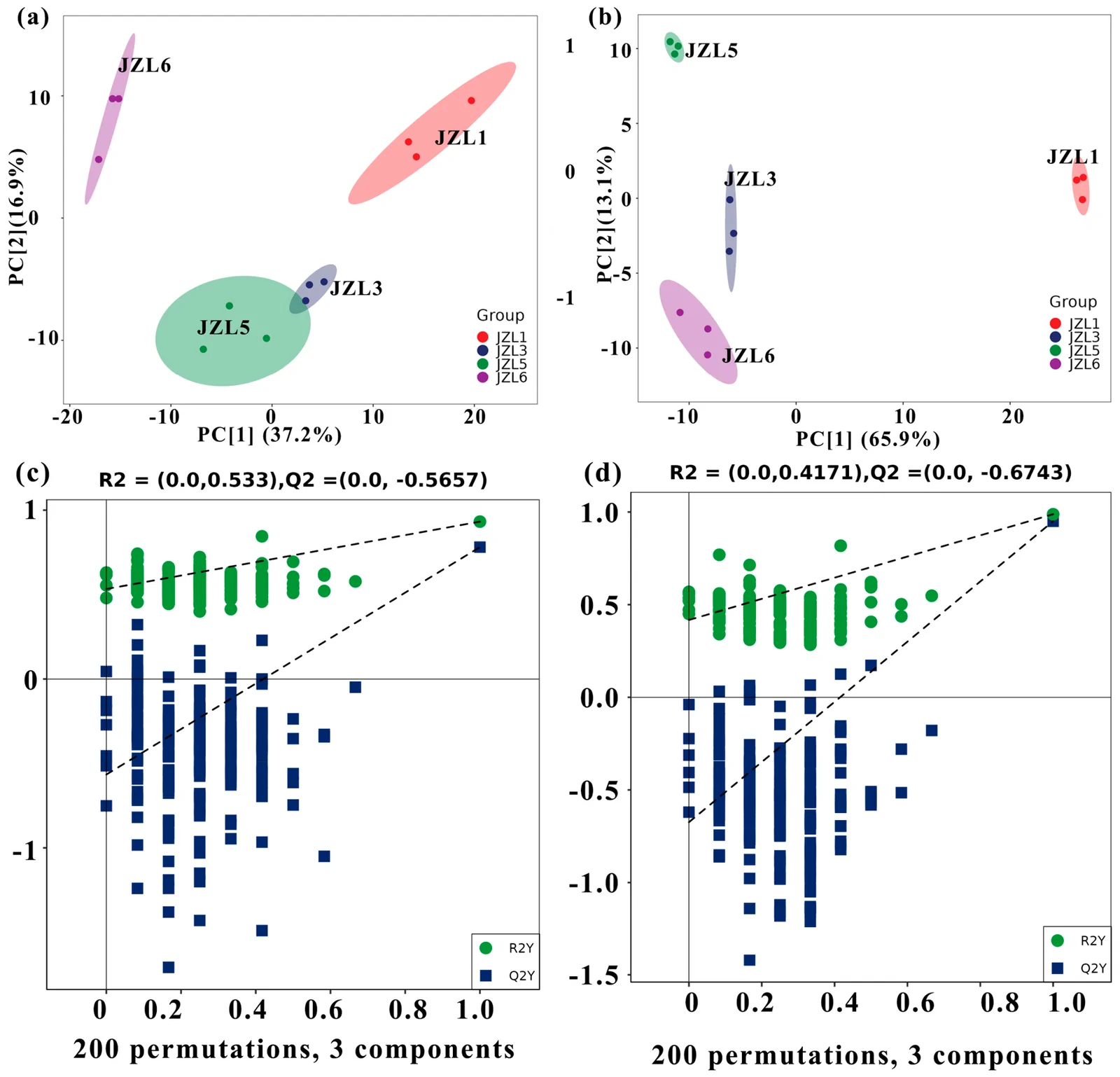
(**a**,**b**) represent the PLS-DA score plots of *Pouteria caimito* fruit metabolites in positive and negative ion modes, respectively. (**c**,**d**) represent the PLS-DA model permutation test plots of *Pouteria caimito* fruit metabolites in positive and negative ion modes, respectively.

The score plots of metabolites from the four *Pouteria caimito* fruit varieties in positive and negative ion modes are shown in Figure 2a and Figure 2b, respectively. In the positive ion mode ion mode, the PLS-DA model yielded R^2^X = 0.571, R^2^Y = 0.857, and Q^2^ = 0.337. In the negative, the PLS-DA model yielded R^2^X = 0.822, R^2^Y = 0.959, and Q^2^ = 0.907. These results indicated that both models exhibited good explanatory power. Meanwhile, the score scatter plots of the *Pouteria caimito* fruit samples showed good clustering, with small intra-group differences, and the four varieties were completely separated, indicating that varietal differences had a significant impact on the metabolite profiles of *Pouteria caimito* fruit pulp.

To avoid overfitting of the fitted PLS-DA models, 200 rounds of cross-validation were performed, as shown in Figure 2c,d. In both models, the Q^2^ regression line crossed the horizontal axis with an intercept below zero, and both R^2^ and Q^2^ were below 1.0, indicating that no overfitting occurred and that the models were statistically significant [20].

### 3.3. Differential Metabolite Analysis of Pouteria caimito Fruit

**Figure 3:**
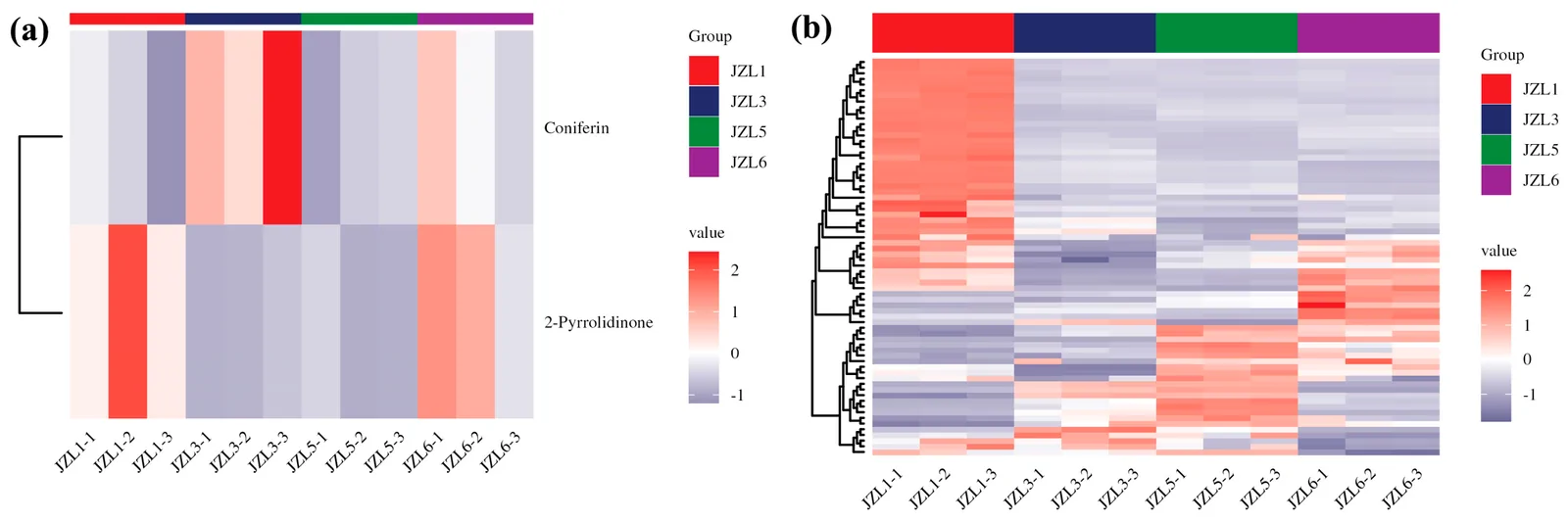

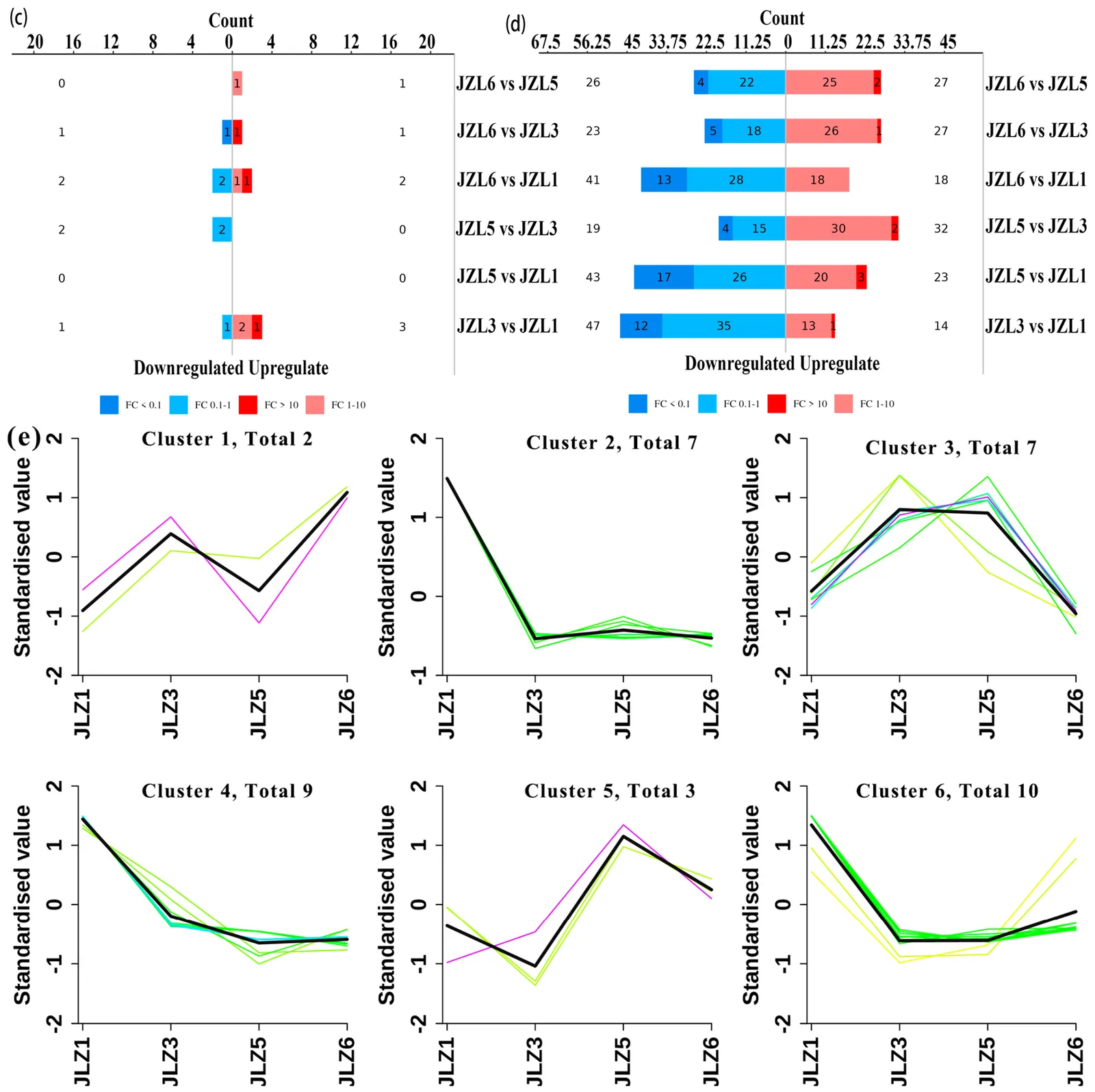
(**a**,**b**) represents the heatmap of differential metabolites of *Pouteria caimito* fruit metabolites under positive and negative ion modes, respectively. (**c**,**d**) represents the number of differential metabolites of *Pouteria caimito* fruit metabolites under positive and negative ion modes, respectively. (**e**) represents the trend of identified metabolites in different groups. The black line represents a simulated trend line, and one line of other colors represents a compound.

As shown in Figure 3a and Table S5, the metabolite profiles of the four cultivars exhibited significant differences, and a total of 72 cultivar-specific differential metabolites were identified. In positive-ion mode, 2 metabolites, including 2-Pyrrolidinone, and coniferin. The content of coniferin was higher in JZL3 than in the other cultivars. In contrast, 2-Pyrrolidinone was enriched in JZL1. As shown in Figure 3b and Table S6, 70 cultivar-specific differential metabolites were identified. In JZL1, a total of 32 metabolites accumulated at higher levels than in the other cultivars, including adenosine, inositol, oryzalin, xanthine, and quinic acid, etc. In JZL3, a total of 2 metabolites accumulated at higher levels than in the other cultivars, including FA 18:1;2O, and Asparagine. Only 12 metabolites, such as trans-11-eicosenoic acid, 2-oxoniocacetate, methyl hexadecanoate, aconitic acid, and 9,10-dihydroxyoctadec-12-enoic acid, etc. showed higher accumulation in JZL5. Similarly, five metabolites, including 8-hydroxyoctadeca-9,12,15-trienoic acid, FA 18:4;O; 9-Hydroxy-10E,12Z-octadecadienoic acid, Linoleic acid, and gamma-linolenic acid and, etc., were preferentially accumulated in JZL6. Among the four varieties, JZL1 had the highest number of varieties-specific differential metabolites, covering multiple chemical classes including nucleosides, sugar alcohols, and organic acids; whereas JZL3 showed the fewest such metabolites and exhibited the smallest compositional differences relative to JZL5. The accumulation of metabolites in tropical fruits is closely related to the activities of metabolic pathways [24], and genetic differences among varieties can directly modulate the expression intensity of corresponding pathways [25,26], thereby affecting the types and abundance distribution of metabolites in the pulp of Pouteria caimito. These differences provide important metabolomic evidence for the quality evaluation and germplasm resource utilization of Pouteria caimito (yellow crystal fruit) cultivars.

As shown in Figure 3c,d and Table S7, in the comparison of JZL3 vs. JZL1, a total of 3 and 14 metabolites were up-regulated in positive and negative ion modes, respectively, including 5 metabolites with fold change > 5, such as 9-Hydroperoxyoctadeca-10,12-dienoic acid, h_92_Oxabolone, 9-Oxo-11-(3-pentyloxiran-2-YL)undec-10-enoic acid, Aspartic acid, and FA 18:1;2O. Meanwhile, 1 and 47 metabolites were down-regulated in positive and negative ion modes, respectively, including 12 metabolites with fold change < 0.1, such as arctigenin, quinate, oryzalin, sorbitol and quinic acid, etc. As shown in Figure 3c,d and Table S8, in the comparison of JZL5 vs. JZL1, 23 metabolites were up-regulated in negative ion modes, respectively, including 6 metabolites with fold change > 5, such as 9-Hydroperoxyoctadeca-10,12-dienoic acid, 9-Oxo-11-(3-pentyloxiran-2-YL)undec-10-enoic acid, 2-Oxonioacetate, (12Z)-9,10-Dihydroxyoctadec-12-enoic acid, Aspartic acid, and cis-Aconitic acid. Meanwhile, 43 metabolites were down-regulated, including 2 metabolites with fold change < 0.1, such as Oryzalin and Arctigenin. As shown in Figure 3c,d and Table S9, in the comparison of JZL5 vs. JZL3, 32 metabolites were up-regulated in negative ion modes, respectively, including 2 metabolites with fold change > 5, such as 2-Azaniumylethyl (2-hydroxy-3-octadec-9-enoyloxypropyl) phosphate, Phosphatidylcholine lyso 18:1. Meanwhile, 2 and 19 metabolites were down-regulated positive and negative ion modes, respectively, including 4 metabolites with fold change < 0.1, such as 3-(4-Hydroxy-3-methoxyphenyl)-2-propenoic acid, N6-Acetyl-L-lysine, Trehalose-6-phosphate, and (-)-Podophyllotoxin. As shown in Figure 3c,d and Table S10, in the comparison of JZL6 vs. JZL1, 2 and 18 metabolites were up-regulated in positive and negative ion modes, respectively, including 5 metabolites with fold change > 5 such as Methionine, 2-Oxonioacetate, Acetanilide, 9-Hydroxy-10E,12Z-octadecadienoic acid, and 8-hydroxyoctadeca-9,12,15-trienoic acid. Meanwhile, 2 and 41 metabolites were down-regulated positive and negative ion modes, respectively, including 13 metabolites with fold change < 0.1, such as (2S,3S,4S,5R,6R)-6-(3-benzoyloxy-2-hydroxypropoxy)-3,4,5-trihydroxyoxane-2-carboxylic acid, QUINATE, 5-O-Feruloylquinic acid, Oryzalin, and Xanthine, etc. As shown in Figure 3c,d and Table S11, in the comparison of JZL6 vs. JZL3, 1 and 27 metabolites were up-regulated in positive and negative ion modes, respectively, including 7 metabolites with fold change > 5, such as FA 18:1;3O, 2-Pyrrolidinone, 2-Azaniumylethyl (2-hydroxy-3-octadec-9-enoyloxypropyl) phosphate, Phosphatidylcholine lyso 18:1, 2-Azaniumylethyl (3-hexadecanoyloxy-2-hydroxypropyl) phosphate, 9,12,13-Trihydroxyoctadeca-10,15-dienoic acid, and Phosphatidylcholine lyso 16:0. Meanwhile, 1 and 23 metabolites were down-regulated in positive and negative ion modes, respectively, including 6 metabolites with fold change < 0.1, such as 9-Hydroperoxyoctadeca-10,12-dienoic acid, Arginine, cis-Aconitic acid, FA 18:1;2O, 9-Oxo-11-(3-pentyloxiran-2-YL)undec-10-enoic acid, and L-Glutathione (oxidized form). As shown in Figure 3c,d and Table S12, in the comparison of JZL6 vs. JZL5, 1 and 27 metabolites were up-regulated in positive and negative ion modes, respectively, including 8 metabolites with fold change > 5, such as Trehalose-6-phosphate, N6-Acetyl-L-lysine, Maleic acid, Gluconic acid, 2-Hydroxy-4-methylvaleric acid, Acetanilide, 3-(4-Hydroxy-3-methoxyphenyl)-2-propenoic acid, and 9,12,13-Trihydroxyoctadec-10-enoic acid. Meanwhile, 26 metabolites were down-regulated in negative ion modes, respectively, including 4 metabolites with fold change < 0.1, such as cis-Aconitic acid, Arginine, 9-Hydroperoxyoctadeca-10,12-dienoic acid, and 9-Oxo-11-(3-pentyloxiran-2-YL) undec-10-enoic acid.

To more intuitively illustrate the variation patterns of key metabolites among the four Pouteria caimito (yellow-fleshed) varieties, metabolites with fold change > 10 or <0.1 were clustered as shown in Figure 3e and Table S13. In Cluster 1, Methionine and Trehalose-6-phosphate showed the highest enrichment in JZL6. Methionine was the sole known precursor for ethylene biosynthesis in plants [27], and its accumulation might indicate a higher ethylene production potential in JZL6, closely linked to fruit ripening and senescence regulation. Trehalose-6-phosphate (Tre6P) was regarded as the “insulin” of plants, acting as a specific sucrose signaling molecule and a central regulator of sucrose levels, integrating metabolic signals with developmental processes; moreover, Tre6P modulates plant growth and metabolism by inhibiting SnRK1 (sucrose non-fermenting-related kinase 1) [28,29]. The strong enrichment of Tre6P in JZL6 suggested that this variety might possess enhanced sugar signaling capacity and carbon allocation efficiency.

In Clusters 2 and 4, metabolites such as Xanthine, Calcium pantothenate, Oryzalin, QUINATE, Raffinose, Hypoxanthine, N6-Acetyl-L-lysine, 3-(4-Hydroxy-3-methoxyphenyl)-2-propenoic acid, Dodecyl benzenesulfonic acid, Feruloyl Hexoxide, and Podophyllotoxin were most enriched in JZL1. Xanthine and Hypoxanthine were key intermediates in the purine degradation pathway, and accumulation of xanthine could activate xanthine dehydrogenase (XDH), which catalyzed the conversion of xanthine to uric acid to scavenge reactive oxygen species (ROS) [30,31]. Raffinose, a representative member of the raffinose family oligosaccharides (RFOs), played critical roles in plant responses to cold, drought, and salt stress [32]. The high enrichment of these metabolites in JZL1 indicates that JZL1 might possess strong stress adaptation capacity and active secondary metabolism.

In Cluster 3, metabolites including cis-Aconitic acid, Arginine, h_92_Oxabolone, 9-Oxo-11-(3-pentyloxiran-2-YL) undec-10-enoic acid, 9-Hydroperoxyoctadeca-10,12-dienoic acid, and FA 18:1;2O were most enriched in JZL3 and JZL5. cis-Aconitic acid was a key intermediate in the tricarboxylic acid (TCA) cycle [33], while Arginine was an important nitrogen storage and recycling amino acid in plants, and also a precursor for the biosynthesis of polyamines, nitric oxide (NO), and other signaling molecules [34]. These two varieties might be more active in polyamine metabolism and nitrogen turnover.

Based on the distribution characteristics of the four clusters, JZL1 showed comprehensive advantages in purine metabolism, osmotic protectants (raffinose, quinate), secondary metabolites (lignans, phenolic glycosides), and coenzyme metabolism, suggesting that JZL1 might be a variety with robust secondary metabolism and strong stress resistance. JZL6 stand out in sugar signaling molecules (Tre6P) and the ethylene precursor methionine, possibly emphasizing sugar signaling and hormonal regulation. JZL3 and JZL5 shared high enrichment of TCA cycle intermediates, arginine, and various fatty acid oxidation products, indicating that these two varieties had similar metabolic profiles in energy metabolism (aerobic respiration) and lipid oxidation signaling pathways, suggesting a relatively close metabolic phenotypic relationship.

### 3.4. KEGG Enrichment Analysis of Differential Metabolites

**Figure 4:**
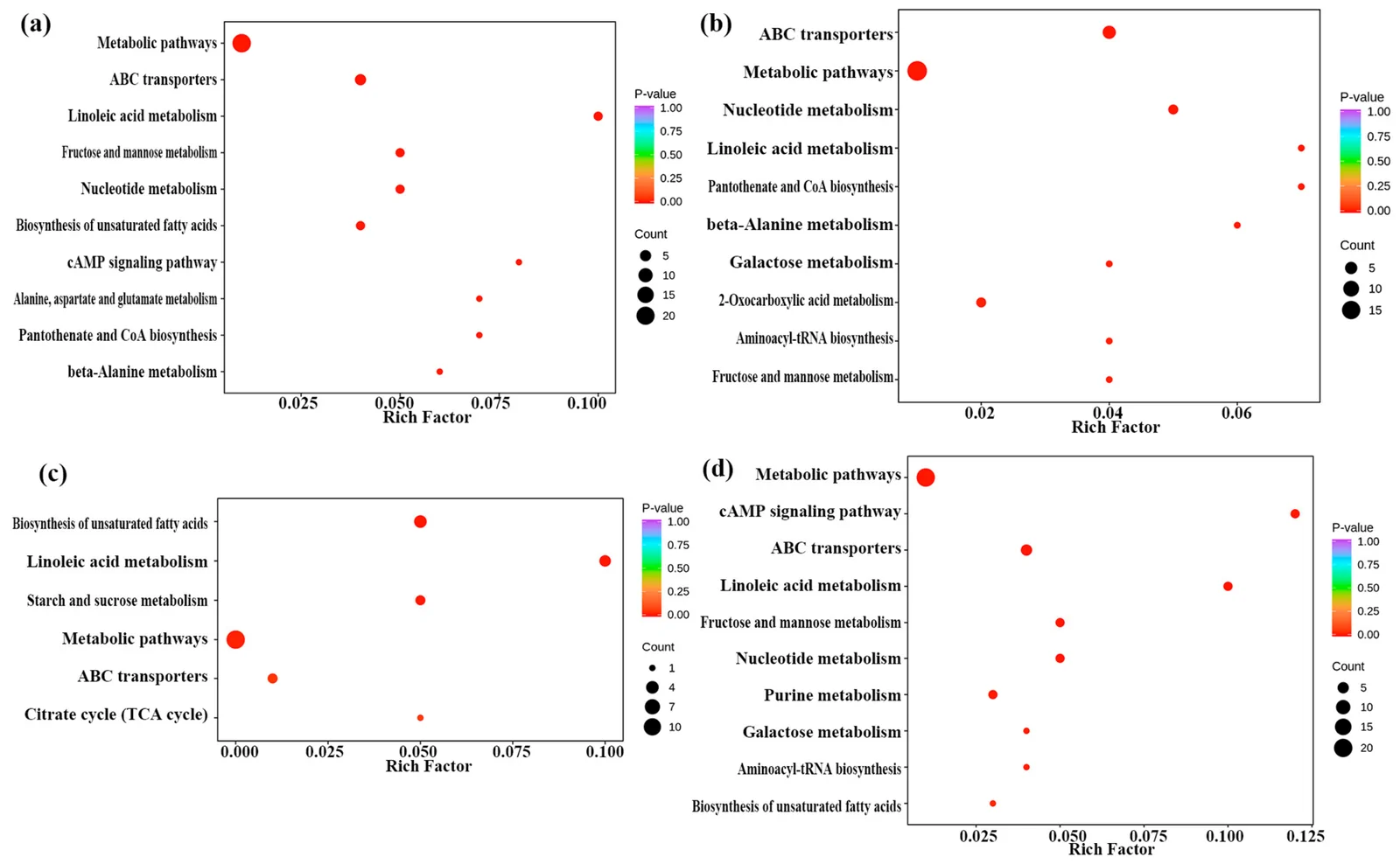

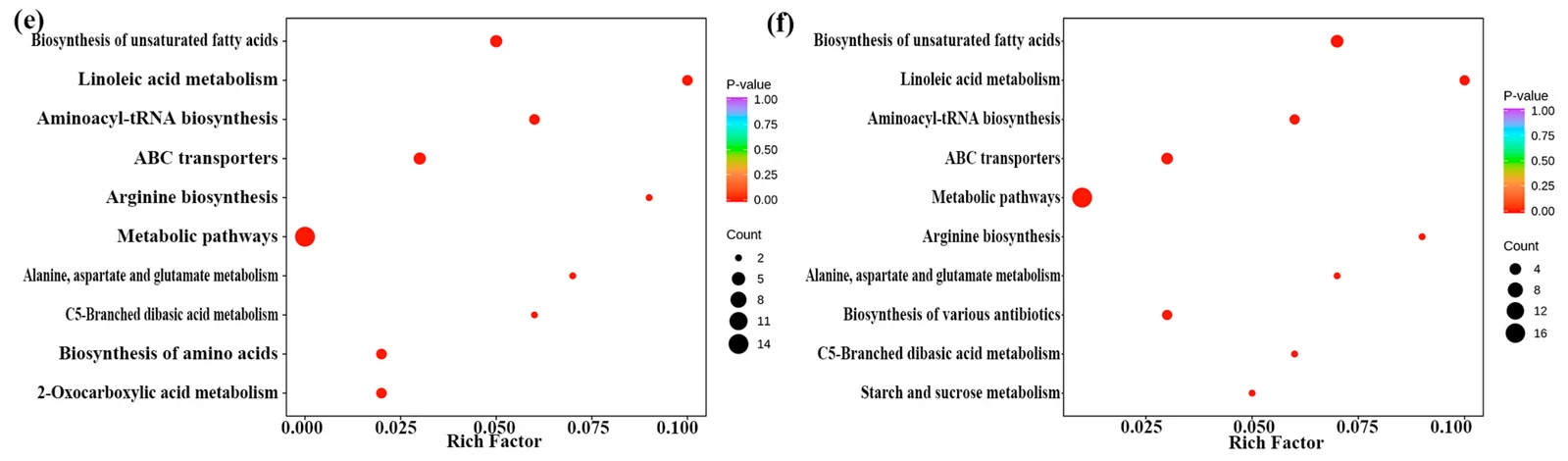
(**a**–**f**) represent the KEGG Pathways enrichment plots for JZL3 vs. JZL1, JZL5 vs. JZL1, JZL5 vs. JZL3, JZL6 vs. JZL1, JZL6 vs. JZL3, and JZL6 vs. JZL5, respectively.

As shown in Figure 4a and Table S14, the differential metabolites in JZL3 vs. JZL1 were mainly enriched in 17 metabolic pathways, including Metabolic pathways, ABC transporters, Linoleic acid metabolism, Fructose and mannose metabolism, and Nucleotide metabolism, etc. As shown in Figure 4b and Table S15, the differential metabolites in JZL5 vs. JZL1 were mainly enriched in 14 metabolic pathways, including ABC transporters, Metabolic pathways, Nucleotide metabolism, Linoleic acid metabolism, and Pantothenate and CoA biosynthesis, etc. As shown in Figure 4c and Table S16, the differential metabolites in JZL5 vs. JZL3 were mainly enriched in 6 metabolic pathways, including Biosynthesis of unsaturated fatty acids, Linoleic acid metabolism, Starch and sucrose metabolism, Metabolic pathways, and ABC transporters. As shown in Figure 4d and Table S17, the differential metabolites in JZL6 vs. JZL1 were mainly enriched in 10 metabolic pathways, including Metabolic pathways, cAMP signaling pathway, ABC transporters, Linoleic acid metabolism, and Fructose and mannose metabolism, etc. As shown in Figure 4e and Table S18, the differential metabolites in JZL6 vs. JZL3 were mainly enriched in 16 metabolic pathways, including Biosynthesis of unsaturated fatty acids, Linoleic acid metabolism, Aminoacyl-tRNA biosynthesis, ABC transporters, Arginine biosynthesis, etc. As shown in Figure 4f and Table S19, the differential metabolites in JZL6 vs. JZL5 were mainly enriched in 19 metabolic pathways, including Biosynthesis of unsaturated fatty acids, Linoleic acid metabolism, Aminoacyl-tRNA biosynthesis, ABC transporters, Metabolic pathways.

Based on the KEGG pathway enrichment analysis, the differential metabolic pathways among the four varieties (JZL1, JZL3, JZL5, JZL6) were summarized in Table 1. As shown in the table, the biosynthesis of unsaturated fatty acids, linoleic acid metabolism, metabolic pathways, and ABC transporters were common to all four varieties. Fatty acid metabolism was not only closely related to fruit membrane lipid composition and cell integrity, but also participates in fruit ripening, senescence, and stress responses [35,36]. As key elements of transmembrane transport, ABC transporters are responsible for the transport of various substances such as sugars, amino acids, and secondary metabolites in plants, and may be involved in the accumulation and distribution of metabolites related to nutrition and flavor in *Pouteria caimito* fruit [37,38]. Therefore, these common pathways might represent the “core modules” of the metabolic network among *Pouteria caimito* varieties and warrant further investigation as potential targets for future quality improvement. Fructose and mannose metabolism, nucleotide metabolism, and galactose metabolism were significantly enriched in JZL1, whereas their enrichment levels were lower or not significant in the other varieties. This finding was highly consistent with the clear separation of JZL1 from the other varieties in the PLS-DA analysis (Figure 2a,c). The composition and proportion of soluble sugars (e.g., sucrose, fructose, and glucose) were key determinants of fruit sweetness [39], fructose and mannose metabolism directly affected the sweetness and energy metabolism of *Pouteria caimito* fruit. Nucleotide metabolism was closely associated with cell proliferation and stress responses [40], while galactose metabolism, as an important component of cell wall polysaccharides, influenced fruit texture [41]. The specific activation of these pathways in JZL1 might indicate that this variety possesses a distinct sugar accumulation strategy or a more active stress-responsive metabolic state.

## 4. Conclusions

In this study, the metabolite composition and differences in the pulp of four *Pouteria caimito* fruit varieties (JZL1, JZL3, JZL5, and JZL6) were analyzed using UHPLC-MS/MS technology. A total of 244 metabolites were identified, covering fatty acyls, prenol lipids, flavonoids, organooxygen compounds, glycerophospholipids, prenol lipids, flavonoids, imidazopyrimidines, isoflavonoids and phenols, etc. PLS-DA analysis indicated that the metabolic profiles of the four varieties were significantly different, and variety was identified as a key factor influencing the metabolite composition of *Pouteria caimito* fruit.

Differential metabolite analysis revealed that a large number of up-regulated and down-regulated metabolites were screened in each comparison group, with some metabolites showing fold changes greater than 10 or less than 0.1 (Methionine, Trehalose-6-phosphate, adenosine, inositol, and oryzalin, etc.), indicating significant differences in metabolite accumulation levels among different *Pouteria caimito* fruit varieties. KEGG enrichment analysis revealed the metabolic pathways involved in the differential metabolites among varieties.

This study not only provided metabolic markers for variety identification of *Pouteria caimito* but also offers an important theoretical basis for subsequent variety-specific quality improvement, functional component development, and postharvest preservation strategy formulation. Future studies may further integrate transcriptomics or targeted metabolomics technologies to deeply validate the metabolites of variety-specific pathways, and to explore their association with fruit quality traits.

## Figures and Tables

**Table 1:** Differential metabolic pathway analysis.

No.	Differential Metabolic Pathways of JZL1 vs. JZL3, JZL5, JZL6	Differential Metabolic Pathways of JZL3 vs. JZL1, JZL5, JZL6	Differential Metabolic Pathways of JZL5 vs. JZL1, JZL3, JZL6	Differential Metabolic Pathways of JZL6 vs. JZL1, JZL3, JZL5
	Pathway Name	Pathway Name	Pathway Name	Pathway Name
1	Metabolic pathways	Biosynthesis of unsaturated fatty acids	Biosynthesis of unsaturated fatty acids	Metabolic pathways
2	ABC transporters	Linoleic acid metabolism	Linoleic acid metabolism	ABC transporters
3	Linoleic acid metabolism	Metabolic pathways	Metabolic pathways	Linoleic acid metabolism
4	Fructose and mannose metabolism	ABC transporters	ABC transporters	Aminoacyl-tRNA biosynthesis
5	Nucleotide metabolism			Biosynthesis of unsaturated fatty acids
6	Galactose metabolism			
7	Aminoacyl-tRNA biosynthesis			
8	Biosynthesis of unsaturated fatty acids			

## Data Availability

The original contributions presented in this study are included in the article/Supplementary Material. Further inquiries can be directed to the corresponding authors.

## Supplementary Materials

The following supporting information can be downloaded at: https://www.mdpi.com/article/10.3390/foods15162855/s1.
